# Roles of Parathyroid Hormone-Related Protein (PTHrP) and Its Receptor (PTHR1) in Normal and Tumor Tissues: Focus on Their Roles in Osteosarcoma

**DOI:** 10.3389/fvets.2021.637614

**Published:** 2021-03-16

**Authors:** Awf A. Al-Khan, Noora R. Al Balushi, Samantha J. Richardson, Janine A. Danks

**Affiliations:** ^1^School of Health and Biomedical Sciences, RMIT University, Bundoora, VIC, Australia; ^2^Department of Pathology, Sohar Hospital, Sohar, Oman; ^3^School of Science, RMIT University, Bundoora, VIC, Australia; ^4^The University of Melbourne, Department of Medicine, Austin Health, Heidelberg, VIC, Australia

**Keywords:** canine, osteosarcoma, parathyroid hormone, parathyroid hormone related protein, prognostic factor

## Abstract

Osteosarcoma (OS) is the most common primary bone tumor and originates from bone forming mesenchymal cells and primarily affects children and adolescents. The 5-year survival rate for OS is 60 to 65%, with little improvement in prognosis during the last four decades. Studies have demonstrated the evolving roles of parathyroid hormone-related protein (PTHrP) and its receptor (PTHR1) in bone formation, bone remodeling, regulation of calcium transport from blood to milk, regulation of maternal calcium transport to the fetus and reabsorption of calcium in kidneys. These two molecules also play critical roles in the development, progression and metastasis of several tumors such as breast cancer, lung carcinoma, chondrosarcoma, squamous cell carcinoma, melanoma and OS. The protein expression of both PTHrP and PTHR1 have been demonstrated in OS, and their functions and proposed signaling pathways have been investigated yet their roles in OS have not been fully elucidated. This review aims to discuss the latest research with PTHrP and PTHR1 in OS tumorigenesis and possible mechanistic pathways.

This review is dedicated to Professor Michael Day who died in May 2020 and was a very generous collaborator.

## Introduction

Osteosarcoma (OS) or osteogenic sarcoma is defined as the malignancy that originates from bone-forming mesenchymal cells (1–5). This tumor is also known as the “growing bone tumor” (6). OS is the primary malignant tumor of the skeleton in which tumor cells directly form immature bone or osteoid (7). OS is the most prevalent type of primary bone cancer in both humans and dogs (8–11). OS occurs more frequently in children, adolescents, taller humans, and large breeds of dogs (9, 12). In both species, OS mostly affects the ends of long bones near the metaphyseal regions (9, 13). The femur, tibia and humerus are the locations that are most often affected by OS in humans (14).

OS is not a modern disease. A recent study revealed that dinosaurs also were affected by OS (15). Ekhtiari et al. confirmed this grossly, radiographically, and histologically in a fibula from a *Centrosaurus* in Canada. The dinosaur dates from around 77 to 75.5 million years ago (15). Previously, paleontologists found periosteal OS using micro-computerized tomography (CT) in the hindleg of a fossilized turtle (16). This was the oldest OS to be found in an amniote indicating that OS was present in this fossil that has been dated to 240 million years old.

There has been little improvement in the treatment of OS and its prognosis in the last 40 years, especially for those patients with metastatic OS (17–21). The reason behind this could be the unavailability of novel biomarkers. Perhaps if there were confirmed prognostic tumor markers, this might assist in categorizing patients for risk-based treatment. Furthermore, the complexity of OS is such that no two tumors look alike (22).

The current treatment strategy for human OS involves neoadjuvant chemotherapy followed by surgical removal of the tumor and adjuvant chemotherapy (23). Standard chemotherapy uses a combination of doxorubicin and cisplatin with a high dose of methotrexate in the neoadjuvant and adjuvant regimens (24). This treatment procedure can improve the five-year survival rate by 60-65% (23, 25). However, early surgical removal of the tumor is the most successful treatment method (26, 27).

Canine and human OS share several key features such as presence of micrometastatic disease at diagnosis, p53 mutations, abnormal expression of several proteins (e.g., activator of transcription 3, tensin homolog, Met, phosphatase, signal transducer and ezrin), affected site and development of chemotherapy-resistance (28). Furthermore, OS in dogs and humans share similar DNA copy number aberrations and show overlapping transcriptional profiles, suggesting that these two diseases are similar at the molecular level. In addition, the metastatic rate of OS without chemotherapy is 90% for dogs and 85–90% for humans and occur mostly in lung, bone and soft tissues, in both species (28).

The high metastasis rate of OS results from the primary bone tumor spread via hematogenous path to other secondary locations (28). The most common cause of death in OS patients is the development of pulmonary metastasis (28). Metastasis occurs most frequently in lungs but rarely occurs in the surrounding pleura. There is one case report where this happened and the authors suggested it was due to the direct contact of pleura with the lungs (29).

Even though <15% of OS metastases in canine and human patients are detected at diagnosis radiologically, 85 to 90% of patients develop gross metastases regardless of effective management of the primary bone tumor (28). This shows that microscopic metastases arise in the early stages of the disease (30). The overall 5-year survival rate for OS in humans is around 60 to 70% in patients with no metastases and 10 to 30% in patients with metastases at diagnosis (24, 31–33). On the other hand, long-term survival rates for OS in dogs is only 10 to 15% (34), supporting the idea that the canine OS may be more aggressive compared to human OS (28).

One study found that overexpression of membrane-cytoskeleton linker ezrin is involved with early development of OS metastases in dogs (35). In line with canine OS data, it has been found that increased expression of ezrin is significantly associated with poor prognosis in OS cases in children (28). Using canine OS cell lines, Hong et al. found that there is an association between PKC and ezrin-radixin-moesin (36). They showed that PKC inhibitor stops ezrin phosphorylation and tumor cell migration (36). Jaroensong et al. reported that overexpression of p-ezrin-radixin-moesin occurred early in the development of pulmonary micrometastases of OS using orthotopic xenograft mouse model of canine OS (37). This expression decreased at later stages suggesting that ezrin is involved in roles related to the survival of cancer cells after their arrival at secondary metastatic sites (37).

Development of metastatic OS is the major cause of death in dogs and humans. So, the identification of new and significant treatments are crucial for the prevention of tumor metastasis which would lead to the reduction of the number of deaths in both dogs and humans (28).

The only basic prognostic indicators of human OS are the patient's response to chemotherapy, the presence of metastases and satisfactory surgical margins (38). Other prognostic indicators such as histological subtype, age, high concentration of serum lactate dehydrogenase or alkaline phosphatase (ALP), tumor size and site are still contentious (38). Recently, it has been shown that the expression of parathyroid hormone receptor 1 (PTHR1) is a prognostic indicator in canine OS (39). Although several studies have been carried out to elucidate the molecular pathogenesis and related signaling pathways of OS using human tissue, murine, canine models and cell lines, the disease remains an unsolved puzzle.

Parathyroid hormone-related protein (PTHrP) was first discovered as a causative factor of humoral hypercalcemia of malignancy syndrome (40, 41). This syndrome occurs because of increased secretion of PTHrP from tumor cells resulting in elevated levels of calcium in serum and increasing cyclic adenosine 3′,5′-monophosphate (cAMP) excretion in urine (42, 43). In humans, PTHrP is synthesized as a protein with either 139, 141, or 173 amino acids due to differences in mRNA splicing (44). PTHrP shares homology of its N-terminal amino acid sequence (1–34) with parathyroid hormone (PTH) (41). This allows both hormones to act through a common receptor (PTH/PTHrP receptor or PTHR1) (45).

PTHR1 is a seven-transmembrane class B G-protein-coupled receptor (GPCR) (46). Examples of receptors included in this family are the receptors for secretin, glucagon, pituitary adenylate cyclase-activating peptide, growth hormone-releasing hormone, vasoactive intestinal peptide, corticotrophin-releasing factor, glucagon-like peptide, calcitonin, and gastric inhibitory peptide (47). Structurally, PTHR1 contains N-terminal extracellular domain (ECD) of ~100–160 amino acid residues, a transmembrane domain (TMD) containing the seven membrane-spanning α-helices and a C-terminal tail (48).

PTHR1 is activated by the binding of the N-terminal (1–34) amino acids of PTH or PTHrP (47). The NH_2_-terminal part of PTH/PTHrP binds to the extracellular connecting loops and the TMD α-helices of PTHR1 (49, 50). This interaction induces conformational changes in PTHR1, which initiates intracellular signaling (51, 52). However, the COOH-terminal part of PTH/PTHrP binds to the N-terminal ECD of PTHR1 (53, 54).

Activation of PTHR1 initiates events of intracellular processes by signaling through the stimulatory G-protein α-subunit (Gsα) (55). Subsequently, the synthesis of cAMP is stimulated and PKA is triggered (56). However, PTHR1 can be activated by another signaling pathway through the Gq class of G-protein α-subunits (Gqα) (57). This activation results in triggering phospholipase C (57) which in turn activates PKC and raises inositol triphosphate and intracellular calcium in tissues (56, 58).

**Figure 1:**
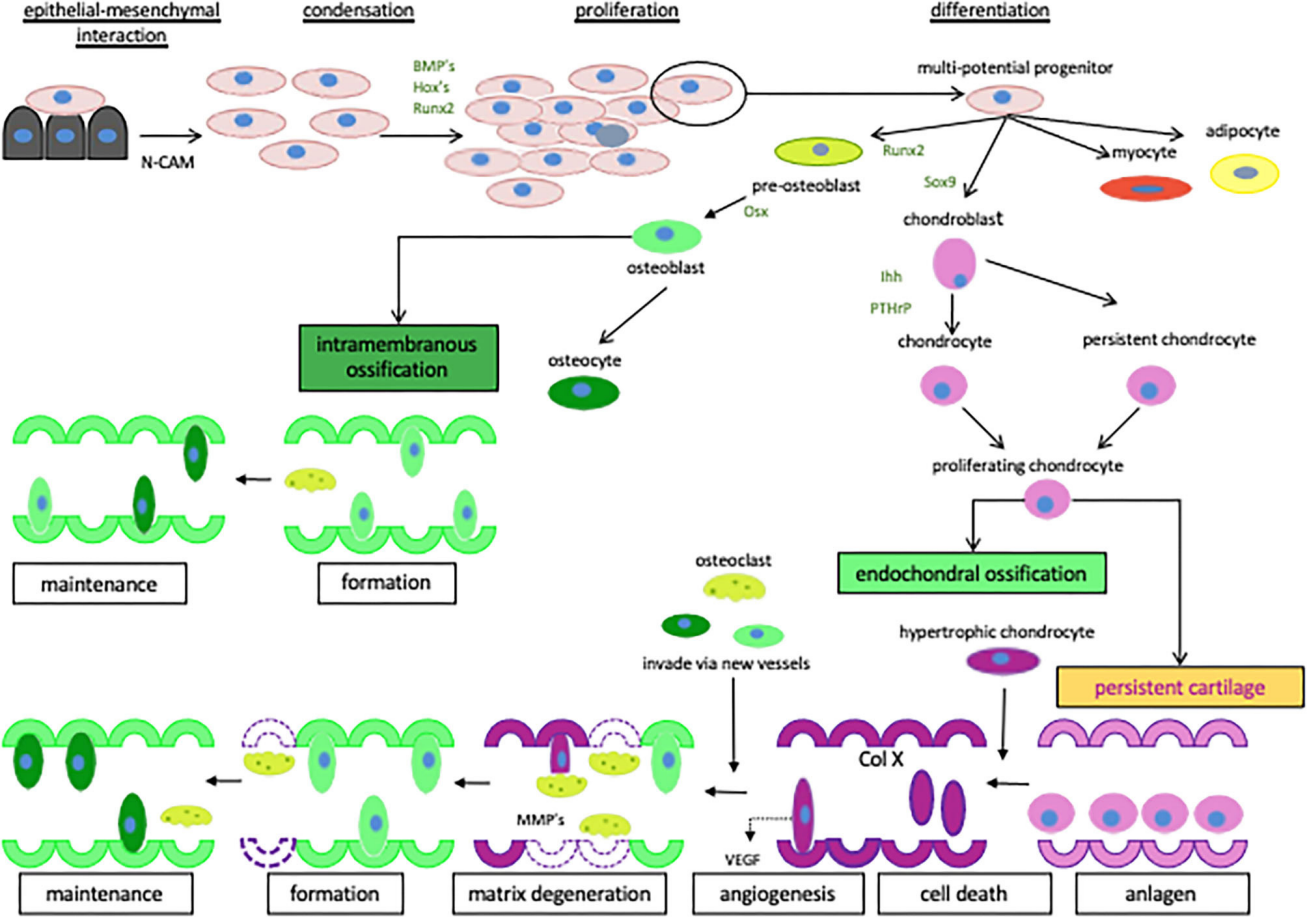
The regulation of cartilage and bone formation. Diagrammatic representation of the network of signaling factors involved in cartilage and bone formation. Starting with the creation of mesenchymal condensations and their subsequent transition to differentiated cartilage and bone. The cells are represented as osteoblasts 
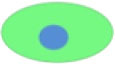
, pre-osteoblasts 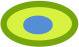, chondroblasts 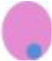, chondrocytes 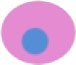, osteoclasts 
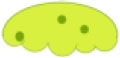
, hypertrophic chondrocytes 
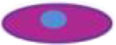
, adipocytes 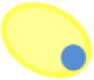 and myocytes 
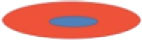
. Reproduced with permission from reference (59).

**Figure 2:**
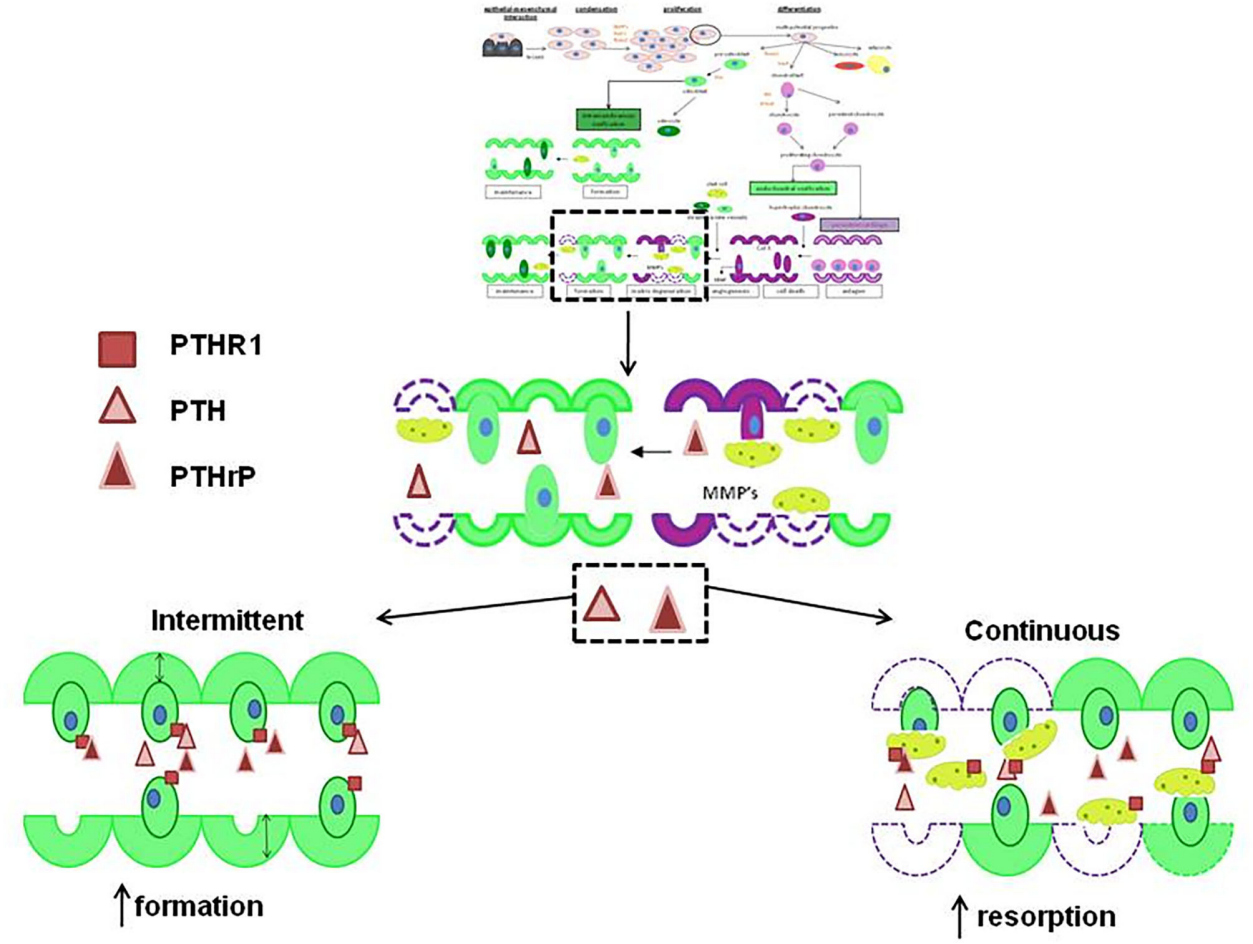
The actions of PTH and PTHrP on new bone formation. If PTHrP 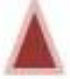 and PTH 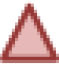 are given intermittently to patients, they increase the formation of new bone 
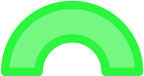
 but if either are given continuously, they increase resorption by stimulating osteoclasts to remodel bone 
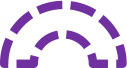
. Both act via PTHR1 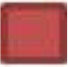. Reproduced with permission from reference (59).

Numerous studies have established the roles of PTHrP and PTHR1 in bone formation, remodeling (Figures 1, 2) and regulation of calcium transport (60–64). In addition, these molecules play a role in the progression and metastasis of many human tumor types such as lung and breast cancers (65, 66). The aim of this review is to highlight the latest findings about functions of PTHrP and PTHR1 in normal and neoplastic tissues by focusing on their roles in the progression of OS and discuss the possible related pathways.

## Roles of PTHrP in Normal and Tumor Tissues

PTHrP acts as an autocrine or paracrine factor and has a role in a number of significant physiological processes in bone, such as the regulation of chondrocyte and osteoblast differentiation and the proliferation (Figure 1) in the growth plates of developing long bones (60, 61). In bone tissue, PTHrP maintains the columnar organization of the chondrocytes and slows down their differentiation (61). Garcia-Martin et al. (67) suggested that PTHrP promotes proliferation of osteoblasts and matrix mineralization via three partially redundant mechanisms. These mechanisms are an intracrine nuclear localization signal-dependent mechanism, an autocrine/paracrine signal-peptide/PTHR1-dependent mechanism, and mixed mechanism (67). Thus, secretion of PTHrP and subsequent activation of PTHR1 would induce proliferation and mineralization of osteoblastic cells (67) (Figure 1).

In addition, PTHrP is involved in significant processes in other tissues including breast (62, 68) and placenta (63, 64). In the breast, PTHrP is abundant in milk, produced via the lactating breast and has an important role in branching morphogenesis of the mammary glands (62, 68). The concentration of PTHrP in plasma is increased during lactation resulting in the regulation of calcium transport from blood into the milk (62) and stimulation of calcium mobilization from bone (68). In the placenta, PTHrP has a role in regulating the direct transport of maternal calcium to the fetus across the placental membrane (63, 64).

Over and above its normal roles, increasing evidence has indicated that PTHrP plays critical roles in tumorigenesis (69–72). It has been found that PTHrP has a role in the activation of protein kinase A (PKA) and C (PKC) pathways (73), regulation of primary tumor growth and in metastasis (72). Luparello et al. (69) found that PTHrP stimulates cell invasion using the 8701-BC human primary breast ductal infiltrating carcinoma cell line. Further data obtained from immortalized human mammary epithelial cell lines (S1T3, S2T2, and NS2T2A1) indicated that PTHrP stimulates proliferation of tumor cells (70). In addition, it has been found that knockdown of PTHrP reduced tumor growth, induced apoptosis of osteoblasts and stimulated the formation of autophagosomes using human MDA-MB-231 breast cancer cell line (74). The authors suggested that blocking of PTHrP in the tumor cells might be a possible targeted therapy for breast cancers, particularly those with skeletal metastases (74). Similarly, Li et al. showed that PTHrP promotes breast tumor initiation, progression and metastasis in mice and it could be a novel therapy target (75). Together, these studies revealed that PTHrP plays a critical role in the initiation of breast cancer (74, 75).

A retrospective study found that increased circulating PTHrP levels might be prognostic with shorter survival time and bone metastases in patients with lung carcinoma (71). Recently, Hastings et al. (65) also examined whether N-terminus or C-terminus of PTHrP correlated with different lung carcinoma type and prognosis. They established that C-terminus of PTHrP may reduce the effect of N-terminus PTHrP on tumor growth and progression (65). Iguchi et al. (76) established the role of PTHrP in bone metastasis in mice models using human lung squamous cell carcinoma-derived cells. Breast and lung cancers usually cause osteolytic metastases in bone (77). This osteolytic process depends on osteoclast-mediated bone resorption via up-regulated osteoclastogenesis (77). Osteoclast differentiation factors, which play a significant role in this process are receptor activator of nuclear factor-jB (RANK), its ligand (RANKL) and the decoy receptor, osteoprotegerin (OPG) (77). In humans, positive PTHrP staining was seen in 60% of primary breast tumors (78) and 92% of bone metastases (79). Recently Kim et al. (66) showed that activation of the calcium-sensing receptor (CaSR), a GPCR, up-regulated the production of PTHrP in breast cancer *in vitro*. As a result, this enhanced proliferation of breast cancer cells and reduced apoptosis (66). It was observed that reducing the expression of CaSR *in vivo* and *in vitro* inhibited the production of PTHrP and reduced the growth of the breast cancer (66).

In addition to breast and lung cancers, PTHrP has been found to stimulate tumor cell survival and proliferation in other cancers including chondrosarcoma (80), anaplastic thyroid cancer (81), medulloblastoma (82), adrenocortical cancer (83), oral squamous cancer (84), colon cancer (85), prostate cancer (86) and renal cancer (87). It has also been found that PTHrP is an essential growth factor for human clear cell renal carcinoma (CCRC) and acts as a novel target for the von Hippel-Lindau tumor suppressor protein *in vitro* (88). Talon et al. (87) demonstrated that apoptosis could be induced in the human CCRC cell line via the induction of PTHrP-neutralizing antibodies followed by the inhibition of PTHR1. Furthermore, Danilin et al. (89) showed that the mRNA-binding protein HuR is involved in increased expression of *PTHrP* and in mRNA stabilization in CCRC. A number of case studies reported a strong expression of PTHrP in pancreatic adenocarcinoma (90), intrahepatic cholangiocarcinoma (91), pancreatic neuroendocrine cancer and that PTHrP levels were elevated in the patient serum (92).

In addition to its role in tumorigenesis, Kir et al. (93) showed that PTHrP is involved in cancer cachexia, a wasting disorder of adipose and skeletal muscle tissues that leads to intensive weight loss resulting in reduced survival time in patients with cancer. PTHrP drives the expression of genes that are involved in thermogenesis in adipose tissue (93). It was demonstrated that the genes responsible for fat and muscle tissue loss were neutralized by anti-PTHrP antiserum (93). In summary, PTHrP is appearing to be a crucial factor in the pathogenesis of a large range of epithelial and non-epithelial tumors.

## Roles of PTHrP in OS

**Figure 3:**
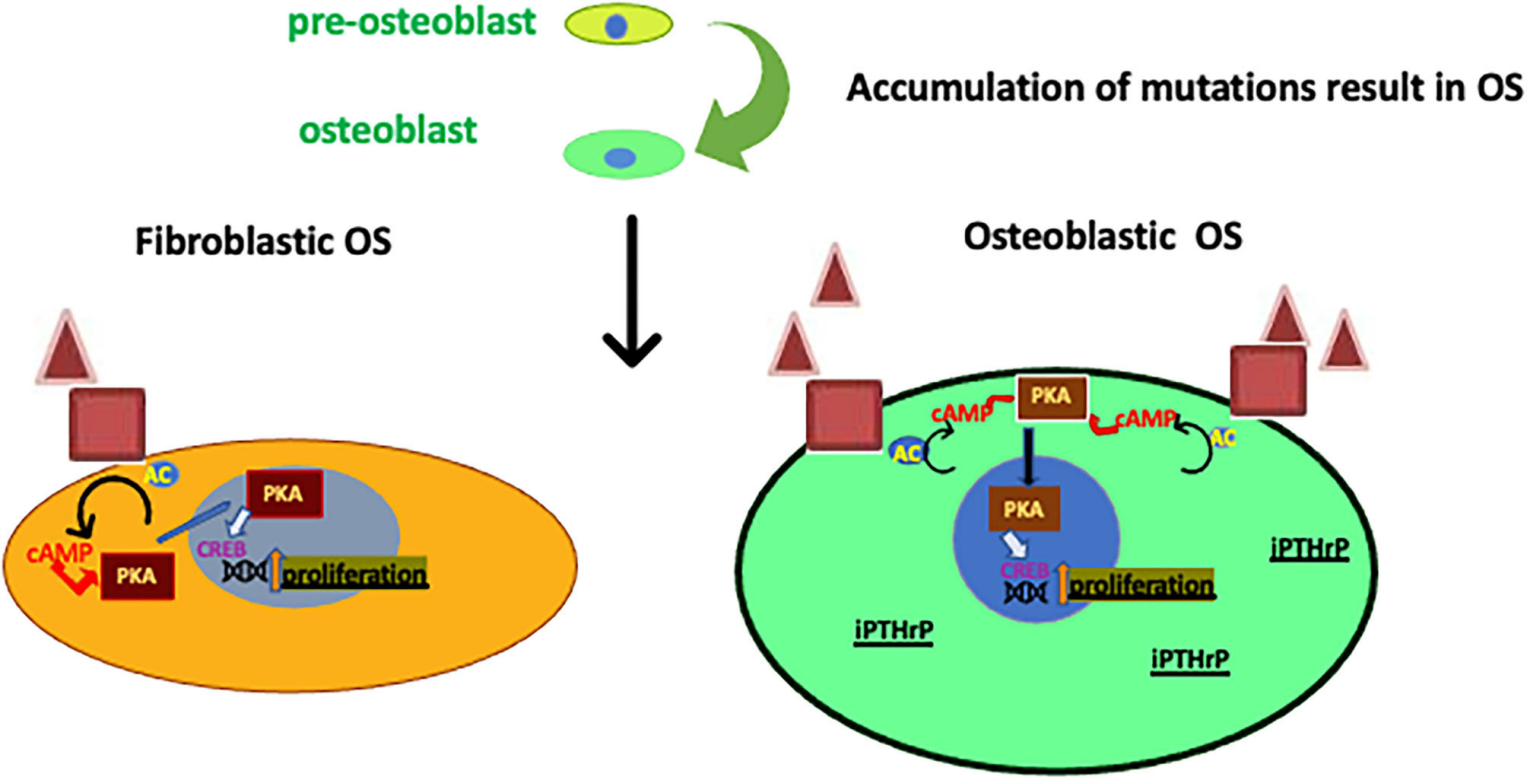
Roles of PTHrP on two of the OS subtypes. Subtypes of OS arise from pre-osteoblasts that accrue mutations (possibly in Rb or p53). PTHrP, PTHR1 and CREB activity are increased in osteoblastic OS influencing proliferation (99, 100) when compared to fibroblastic OS. Also, the intracrine PTHrP (iPTHrP) may contribute to this process.

The first attempt to understand the role of PTH in OS was by Martin et al. (94) by inducing OS in rats using radioactive phosphorous isotopes. Several later studies found that PTHrP also plays a role in pathogenesis of OS (Table 1, Figure 3) (96, 101–104). Suda et al. (102) demonstrated the expression of PTHrP mRNA in all investigated rat UMR 106-01 and UMR 106-06 OS cell lines. Ho et al. (99) revealed that PTHrP is also expressed by murine OS cells. Recently, PTHrP was detected in all primary canine OS tissues (*n* = 50) using immunohistochemistry staining (39). The findings showed that 50% of these canine OS tissues had weak staining intensity and 50% strong staining intensity. The study also found that there was not significant correlation between the staining intensity and the prognosis of OS in dogs (39).

**Table 1:** Roles of PTHrP in progression of OS.

**Role of PTHrP in OS**	**Type of tissue**	**Species**	**References**
Increased expression of *PTHrP* is associated with reduced tumor growth and cell proliferation	Cell line	Rat	(95)
Increased expression of *PTHrP* is correlated with decreased cell proliferation and tumor growth	Cell line	Mouse	(96)
Overexpression of *PTHrP* caused tumor chemoresistance	Cell line	Human	(97)
Overexpression of *PTHrP* stimulates migration of tumor cells	Cell line	Human	(98)
Inhibition of *PTHrP* reduced cell growth and invasion	Cell line	Mouse	(99)
Knockdown of *PTHrP* increased apoptosis and growth inhibition	Tissue	Mouse	(99)
Presence of PTHrP protein in tumors was not a prognostic marker	Tissue	Dog	(39)

In fact, the immunohistochemical (IHC) staining of PTHrP demonstrated the presence of the protein in the OS at the time of staining, but it does not tell us how much PTHrP is produced and secreted over the time (39). This association between the presence of PTHrP protein and prognosis has not yet been investigated in humans

**Figure 4:**
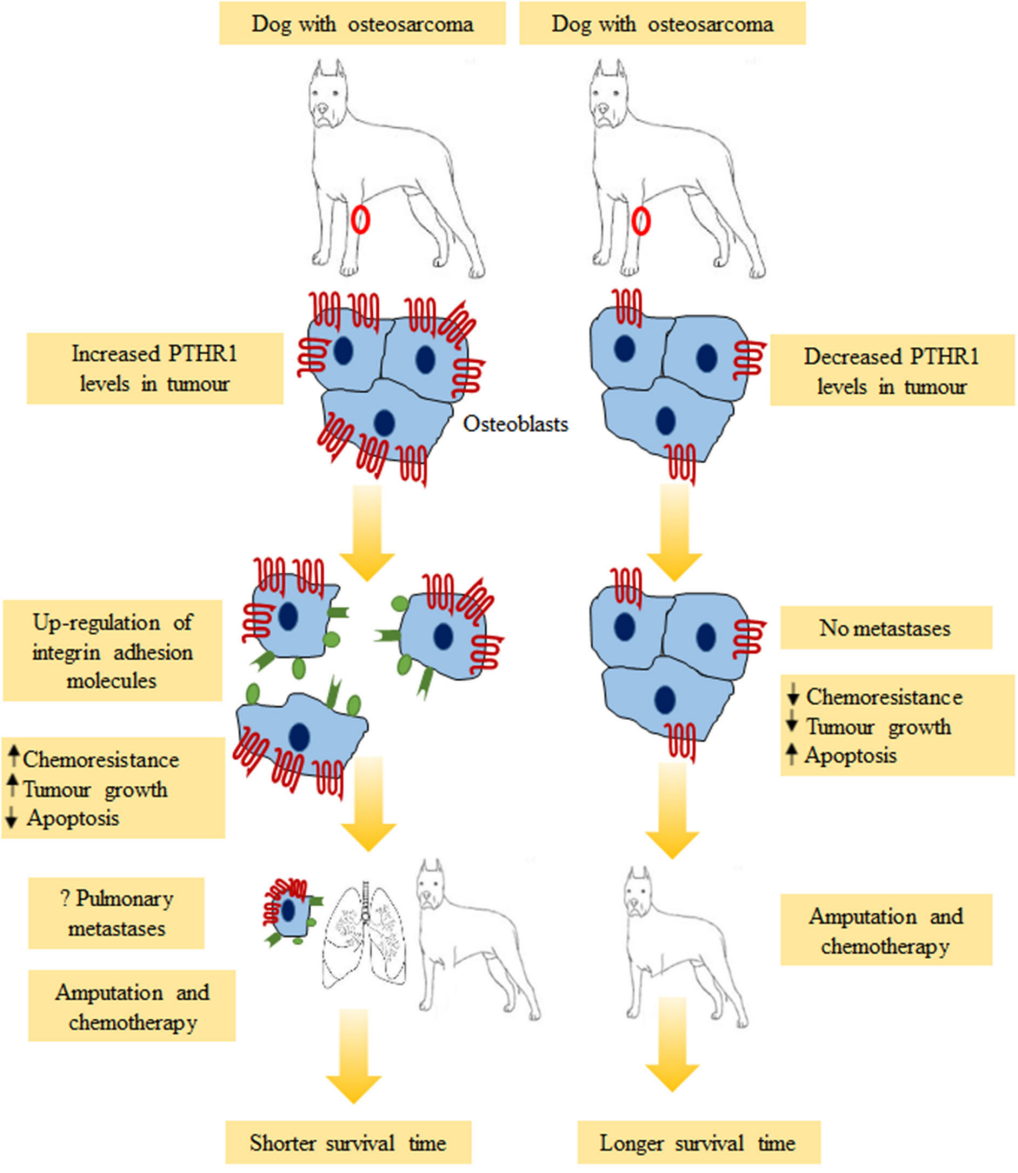
Possible outcomes for dogs with osteosarcoma. Dogs with strong PTHR1 immunostaining tumors had shorter overall survival times compared to those with weak immunostaining. Overabundance of PTHR1 could activate neoplastic osteoblasts to detach via up-regulation of integrin adhesion molecules (αvβ3, β1, α2β1, α5β1, α6β1), resulting in pulmonary metastases. Other possible mechanisms which could explain the effects of PTHR1 expression including increased chemoresistance, increased tumor growth and decreased apoptosis. This might result in shorter survival time.

In contrast, PTHrP mRNA was not detected in aggressive human OS xenografts (105). It has also been found that increased expression of the *PTHrP* gene is associated with reduced tumor growth and cell proliferation (Table 1) using a murine OS cell line (96) and a rat OS cell line (106). Previous findings discussed above showed that over-expression of *PTHrP* could be correlated with a better prognosis for OS (Figure 4).

However, Gagiannis et al. (97) noted that PTHrP caused tumor cells of SaOS2 human OS cell line to be chemoresistant (Table 1). This was observed after inhibiting major apoptosis signaling pathways via blocking the death receptor and mitochondria-mediated apoptosis signaling (97). It has also been found that PTHrP stimulates migration of SaOS-2 and MG-63 human OS cell lines (98). These two studies suggest that overexpression of PTHrP could be correlated with a poorer prognosis of OS (97, 98). These conflicting data may be related to the use of different portion of PTHrP sequences in these different studies (67, 107). If PTHrP influences chemoresistance then it would be a good therapeutic target. Blocking this action could improve patient survival with current treatments.

Ho et al. (99) found that the three major OS subtypes (osteoblastic, chondroblastic and fibroblastic OS) produce PTHrP, which act through the PTHR1 to activate adenylyl cyclase, PKA, and the transcription factor cAMP responsive element binding protein 1 (CREB1) (Figure 3) (99). The osteoblastic subtype had an increased level of PTHR1 compared with the fibroblastic subtype but the PTHrP levels were no different (99). The knockdown of PTHrP in OS reduced cell growth and invasion *in vitro* and increased apoptosis and growth inhibition *in vivo*, while the knockdown of CREB1 had much greater growth inhibition and apoptosis (99). Moreover, Walia et al. (108), found that PTHrP is a key factor for initiation of OS in p53-deficient osteoblasts. The production of cAMP is stimulated by PTHrP (108). This stimulation is followed by PTHR1 activation, then, phosphorylation and transcription of CREB1 is activated in p53-deficient OS (Figure 3) (108). It was suggested that PTHrP-cAMP-CREB1-axis is essential for the initiation and progression of OS in p53-deficient osteoblasts (108). These findings are significant because P53 deficiency is a common event in OS and understanding of this pathway could lead to a better elucidation of this disease (108).

All of the above data showed that PTHrP is crucial for tumorigenesis of OS and increased expression could be linked with poor prognosis in mice (Table 1). However, further *in vivo* studies are necessary to clarify the exact roles of PTHrP in the progression of OS, possibly to be undertaken in dogs.

## Roles of Human Parathyroid Hormone in OS

The active portion of human parathyroid hormone is a 34-amino acid peptide (109). Studies demonstrated that PTH (1–34) and the native 84-amino acid hormone have identical spectrum of biological responses in bone (110, 111). It has been shown that single-daily subcutaneous administration of PTH (1–34) accelerates the production of new bone matrix on the endocortical, trabecular and periosteal surfaces via the stimulated osteoblasts (Figure 2) (110). This leads to significant elevation of bone mineral density, bone mass and strength of the bones (112, 113). Because of this, PTH (1–34) or teriparatide has been used in the management of adult patients with osteoporosis to increase bone mass and prevent bone fracture (114–117).

The Food and Drug Administration (FDA) approved teriparatide Eli Lilly & Co. (Indianapolis, IN, USA) as a treatment for osteoporosis under the name “Forteo” in November 2002 (118). The approval of this drug came after preclinical and clinical trials produced some conflicting results. Data from preclinical trials revealed that a high number of rodents developed OS after the treatment with very large doses of teriparatide for most of their lifespan. For this reason, the FDA was required to balance the possible side effects with the vital benefits of this distinctive product (118). In addition, teriparatide is not used to treat patients affected by primary malignant and metastatic bone tumors (119), Paget's disease (120) or who have had radiotherapy (121). All these conditions may increase the probability of OS development in patient treated with teriparatide (122).

Watanabe et al. showed that the induction of OS in rats treated with teriparatide depends on the duration and dose of treatment (123). In 2004, Vahle et al. described a safe regime of teriparatide for rats (124), starting with 5 μg/kg at 6 months of age and continued for either six or 20 months (up to 70% of life span) resulted in significant increase in bone mass with no development of neoplasms (124).

In humans, two cases of OS after treatment with teriparatide have been reported in the USA (122). Nevertheless, in the first case, the connection between teriparatide and the OS was not clear (121). In the second case, the patient was treated with radiotherapy before treated with teriparatide; thus, it is uncertain whether the teriparatide treatment or radiotherapy was associated with development of OS (119). Recently, another patient developed OS after administration of teriparatide (122). This patient had no history of Paget's disease and had never received any radiotherapy. According to Ogawa et al. (122), this case was the first case with definite correlation between teriparatide and acceleration of growth of a pre-existing malignant tumor in humans.

Hyaluronan (HA) is a glycosaminoglycan component of the extracellular matrix. It is involved is regulation of cancer cell function (125, 126). It has been found that PTH increases the production of HA in osteoblast-like OS cell line (UMR 106-01 BSP) (127). Furthermore, as a response to PTH, endosteal and periosteal osteoblastic cells exhibited metabolic variances in their HA synthesis (128). It is suggested that PTH (1–34) has a role in an administration mode-dependent manner, on HA metabolism that is vital for migration of OS cell (98). This role is correlated with OS cell differentiation and behavior (98). Treatment of aggressive and poorly differentiated MG-63 cells with intermittent PTH (1–34) was found to increase expression of their HA-synthase-2, which lead to enhanced high-molecular size HA deposition in the pericellular matrix and increased migration of these cells. Continuous treatment of well-differentiated Saos2 cell with PTH (1–34) also increased the production of HA and modestly stimulated their migration (98). Another study showed that the anabolic effect of PTH (1–34) on bone metabolism was associated with changes in fibroblast growth factor-2 (FGF-2) expression (129). These FGF variations could modify the nuclear accumulation and subsequent action of runt-related transcription factor 2 (Runx-2) and CREB transcription factors which are important in the regulation of osteoblast differentiation and growth (129).

Although the mechanism responsible for the rodent bone neoplasms is still a puzzle, it was suggested that the incidence of bone tumors is increased as a result of the prolonged treatment period in these rats in conjunction with an extreme response of the skeleton to the elevated bone formation effect of daily administration of teriparatide (110). Moreover, as mentioned previously, PTHR1 is activated by the binding of the N-terminal (1–34) amino acids of PTH or PTHrP (47). The abundant production of PTHrP which can bind to PTHR1 and promote the formation of cAMP could result in induction of OS as it will be discussed in the section “Roles of PTHR1 in OS” (130). Hypothetically, treatment with teriparatide and blocking of PTHR1 at the same time could reduce the possibility of OS induction. More studies are warranted to clarify the correlation between PTH, PTHR1, and OS.

## Roles of PTHR1 in Normal and Tumor Tissues

PTHR1 is found mainly in bones and kidneys (131), and is involved in mineral ion homeostasis, bone turnover and skeletal development (132). In bone, PTHR1 regulates function, differentiation and proliferation of chondrocytes and osteoblasts (Figure 1) (133–135). It also controls calcium release from the matrix (136, 137).

In the kidney, PTHR1 has a role in the reabsorption of calcium in the distal convoluted tubule (46, 138) and in the maintenance of blood phosphate levels via inhibiting phosphate reabsorption in the distal and proximal tubules (139, 140). It also increases the activity of 1α-hydroxylase, resulting in increased calcium absorption from the intestine through increasing levels of 1,25-dihydroxycholecalciferol (46, 138).

Expression of PTHR1 protein has been detected in human primary tumors, including melanoma (100%), prostate adenocarcinoma (100%), colorectal carcinoma (100%), OS (50%), renal cell carcinoma (23%), and breast carcinoma (17%) (141). Studies showed that expression of PTHR1 was also detected in several human breast cancer cell lines (70, 142). Previously, Linforth et al. (143) found that expression of PTHR1 is correlated with poor prognosis in patients with primary breast cancer whilst Hoey et al. (144) reported that PTHR1 was highly expressed in human breast cancer bone metastases samples compared to primary breast cancer. The overexpression of PTHR1 in MCF-7 cells stimulated tumor cell proliferation through autocrine signals, which are mediated by cAMP and extracellular signal-regulated kinase (ERK) pathways (144).

In addition to PTHR1, recent studies have shown that overexpression of other GPCRs were associated with poor prognosis in pancreatic, breast and prostate cancers (145–147). Li et al. (146) found that increased expression of purinergic receptor *P2Y2*, a class A GPCR, correlated with a poor prognosis in prostate cancer. Moreover, protease-activated receptor 1 (*PAR1*), a second-class A GPCR, was reported to be highly expressed in aggressive breast tumors (146). Wang et al. (147) found that overexpression of GPR87, another class A GPCR, was linked with reduced survival for patients with pancreatic cancer. Furthermore, *GPR87* was reported to promote aggressiveness in primary cell lines derived from the above patients' tumors (147). These data might support the carcinogenicity of PTHR1 and other GPCRs.

PTHR1 was not well-studied in cancers other than breast and OS. The next section highlights the critical roles of PTHR1 in OS.

## Roles of PTHR1 in OS

Numerous studies using human cell lines (105), murine (99), human (141) and canine (39) tissues have reported the association between overexpression of PTHR1 and OS progression (Table 2). Mutsaers et al. (100) detected PTHR1 in primary and metastatic OS of osteoblastic and fibroblastic subtypes *in vivo* from two different types of transgenic mice. It has been suggested that increased expression of PTHR1 in OS could stimulate progression by formation of a more aggressive subtype (105).

**Table 2:** Roles of PTHR1 in progression of osteosarcoma.

**Role of PTHR1 in OS**	**Type of tissue**	**Species**	**References**
Overexpression of *PTHR1* is linked with increased invasion and proliferation	Cell line	Human	(105)
Knockdown of *PTHR1* stimulated tumor differentiation and decreased invasion and growth	Tissue	Mouse	(99)
Blocking of PTHR1 reduced metastatic cell invasion, proliferation, migration and adhesion	Cell line	Human	(148)
Patients with strongly staining for PTHR1 OS tumors had reduced survival times compare to those with weak immunostaining intensity OS tumors	Tissue	Dog	(39)
Decreased mRNA expression of *PTHR1* inhibited proliferation, migration and invasion	Cell line	Human	(149)

*PTHR1* mRNA is highly expressed in metastatic human OS compared with primary tumors (105). Overexpression of *PTHR1* was linked with increased invasion and proliferation in 143B, U2OS, SaOD-2 and HOS cell lines (105). In addition, Ho et al. (99) reported that knockdown of *PTHR1* in murine OS cells stimulated tumor differentiation and decreased invasion and growth. It has been found that reduced expression of PTHR1 *in vivo* enhanced mineralization and differentiation in OS (99).

Recently, immunostaining for PTHR1 was detected in all canine OS tissues (n = 50) (39). The findings showed that dogs with PTHR1 strongly staining OS tumors had significant shorter survival time compared to those with weakly staining tumors (39). According to this study, dogs with appendicular OS showing PTHR1 strong immunostaining lived for 212 days compared to those with weak immunostaining who lived for more than double the time (459 days). The conclusion was that expression of PTHR1 could be a significant prognostic indicator in canine OS (39). As was mentioned previously, the relationship between the expression of PTHR1 and survival time of OS patient has not yet studied in humans. However, recent experiments by the group at Liaoning Cancer Hospital showed that treatment of human Saos-2 and U2OS cell lines with mangiferin, a xanthonoid, decreased mRNA expression of *PTHR1 in vitro* (149). This study suggested that the inhibition of proliferation, migration and invasion of OS cells that resulted from this treatment are correlated with inhibition of PTHR1 (149). Moreover, a recent evidence revealed that blocking of PTHR1 in human Saos-2 and U2OS cell lines by using of Quercetin, a flavonoid found in vegetables, fruits, and grains, reduced metastatic cell invasion, proliferation, migration, and adhesion (148). These findings suggest that PTHR1 could be a novel and promising therapeutic target for OS.

The pathway of PTHR1 in tumorigenesis of OS was suggested by Walkley et al. (130). Under normal conditions, PTHrP binds and activates PTHR1which is located on the surface of osteoblasts. Activation of PTHR1 leads to the synthesis of cAMP from ATP via adenylyl cyclase. Consequently, cAMP induces the detachment of cAMP-dependent PKA from its α regulatory subunit of PKA type 1 (PRKAR1A) (130). Activated PKA translocates into the nucleus to phosphorylate and activates CREB. As a result, target genes downstream of *PTHR1* signaling are activated (130). In OS, several abnormalities in the PTHrP-PTHR1-PKA pathway increased the activity of PKA pathway. This includes an elevated number of PTHR1 on the cell surface and increased expression of the *Prkaca* gene that encodes the catalytic component of PKA (130). Other abnormalities are increased production of PTHrP, which can bind to PTHR1 and promote the formation of cAMP and mutations in *PRKAR1A* gene, which result in an increase in the PKA activity (130).

A recent study carried by Li et al. (150) proposed that the effects of PTHR1 could be mediated by angiogenesis, inflammation and the Wnt pathway through altering the expression of the crucial enriched genes (*Dkk1, Lef1, Agt-CCR3*, and *Agt-CCL9*) using mouse OS cells.

Previous studies have reported that integrin adhesion molecules are involved in the migration of OS cells (151–153). Up-regulation of integrins including α5β1, α2β1, α6β1 (151), β1 (152) and αvβ3 (153) was associated with aggressive metastastic OS. PTHR1 could have a role in down-regulation or up-regulation of cell-cell or cell-extracellular matrix adhesion molecules. Integrins might be upregulated by PTHR1 in aggressive OS (Figure 4). To validate the current hypothesis and to further understand OS, future studies should investigate the correlation between PTHR1 and integrins in OS.

The results from all these studies taken together, show that detection of PTHR1 in OS could predict prognosis and therefore may be a potential therapeutic target.

The obvious question that may arise from this review is, why increased immunostaining of PTHR1 is correlated with reduced survival time, although dogs studied by Al-Khan et al. (39). had no clear evidence of metastasis at presentation in the smaller group (*n* = 20 dogs). This suggests that increased amounts of PTHR1 may activate tumor cells later to detach and metastasize to the lung, which leads to a reduced survival time (see Figure 4). The increase in PTHR1 in OS could be correlated with increasing the capability of tumor cells to metastasize and this was supported by a recent study (99). Knockdown of *PTHR1* in OS reduced invasion of tumor cells *in vitro* (99). In addition, Yang et al. (105) revealed that overexpression of *PTHR1* increased invasion and showed that metastatic OS had increased expression of *PTHR1* mRNA compared to the primary tumor.

## Cytoplasmic and Nuclear Localization of PTHrP and PTHR1 in OS

It has been found that full length PTHrP has a nuclear localization signal (NLS) that allows transport into the nucleus after binding to the transport regulatory protein, importin β in the cytoplasm (154). PTHR1 binds to both importin α1 and importin β (155). PTHR1 overexpression has been found in the nucleus during early interphase stage (G0/G1, S, and G2 phases) of the cell cycle in the following cell lines; SaOS-2 human OS, MC3T3-E1 mouse non-transformed osteoblasts and ROS 17/2.8 rat OS (155). At G0/G1, S, and G2 phases, DNA is more open to transcriptional activity compared to the later phases where DNA is compact, transcriptional activities are reduced and the immunofluorescent staining of PTHR1 was weaker (155).

The localization of PTHrP was observed in the cytoplasm of canine primary OS cells in 66% cases and in the nucleus plus the cytoplasm in 34% cases (*n* = 50 dogs) (39). Similarly, PTHrP was detected in the cytoplasm and nucleus using murine OS tissue (99) and human metastatic bone lesions in patients with prostate carcinoma (156). In contrast, PTHR1 was localized to the cytoplasmic plus nucleus of canine OS cells in 100% cases. Another study detected PTHR1 in the cytoplasm of murine OS cell (99), while it was detected also in the nucleus and cytoplasm of normal rat liver cells (157). The study of Al-Khan found that there was no significant correlation between the localization of PTHrP and PTHR1 and prognosis of OS in dogs (39). According to this study, the increased nuclear localization of PTHR1 in OS cells could be linked to the high rate of mitosis. Moreover, most of these cells are at stage G0 and G1.

On the other hand, it has been found that nuclear localization of PTHrP is correlated with inhibition of apoptosis using nine human and rat prostate cancer cell lines [PC-3, PC-3 MB, LNCaP, DU-145, AT-2.1, MLL, AT-3.1, MAT-Lu (ML), and GP9F3] (156). It is suggested that PTHrP has a vital role in the promotion of prostate tumor growth and/or progression (123). Another study revealed that nuclear localization of PTHrP promotes survival of chondrocytes under conditions that stimulate cell death using COS-7 cell line (158).

The only study that investigated the immuno-localization of PTHR1 in human OS cells did not mention the pattern of the immunostaining and they used only four cases of OS (141). The study of Al-Khan et al. (39) is the only immunohistochemical study that investigated the localization of PTHR1 in canine OS. More studies are warranted to confirm the present findings.

## Conclusion

In conclusion, this review has shown that canine OS is a good model for the human disease and highlighted the roles of PTHrP and PTHR1 in normal tissue and in OS. Both PTHrP and PTHR1 are crucial factors for induction of OS. Increased expression of these two proteins in OS is correlated with a poor prognosis. PTHrP and PTHR1 play critical roles in pulmonary metastasis, chemoresistance, tumor growth and decreased apoptosis in OS patients. Although the function of these two proteins in bone, breast, placenta, and kidney has been described, their evolving roles in the pathogenesis of OS requires further investigation. This review supported the proposition that PTHR1 could be a novel and significant prognostic indicator in OS and both PTHrP and PTHR1 could be targets for novel therapeutics for OS. Also, future studies on the correlation between increased expression of PTHR1 and integrins may improve our understanding of OS progression via the discovery of novel signaling pathways that could be manipulated to improve patient outcomes.

## Author Contributions

AA-K reviewed the literature and wrote the manuscript. JD, SR, and NA wrote and edited the manuscript. All authors read and approved the final version of the manuscript.

## Conflict of Interest

The authors declare that the research was conducted in the absence of any commercial or financial relationships that could be construed as a potential conflict of interest.
